# Factors associated with repeat rectal *Neisseria gonorrhoeae* and *Chlamydia trachomatis* screening following inconclusive nucleic acid amplification testing: A potential missed opportunity for screening

**DOI:** 10.1371/journal.pone.0226413

**Published:** 2019-12-12

**Authors:** Cheríe S. Blair, Omai B. Garner, Bettina Pedone, Sam Elias, W. Scott Comulada, Raphael J. Landovitz

**Affiliations:** 1 Department of Medicine, Division of Infectious Diseases, David Geffen School of Medicine at UCLA, Los Angeles, California, United States of America; 2 Department of Pathology and Laboratory Medicine, David Geffen School of Medicine at UCLA, Los Angeles, California, United States of America; 3 Arthur Ashe Student Health and Wellness Center, University of California, Los Angeles, California, United States of America; 4 Department of Psychiatry and Biobehavioral Services, University of California, Los Angeles, California, United States of America; Emory University School of Medicine, UNITED STATES

## Abstract

**Objective:**

Given rising incidence of *Neisseria gonorrhoeae* and *Chlamydia trachomatis* (GC/CT), development of efficacious screening strategies is critical to interruption of the infection cycle. However, a small proportion of nucleic acid amplification testing (NAAT) results are inconclusive—resulting in delays in diagnosis and treatment. As such, this study seeks to evaluate factors associated with inconclusive rectal GC/CT NAAT.

**Methods:**

This is a retrospective chart review of individuals who received an inconclusive rectal GC/CT NAAT result at a single institution from 3/2016-6/2018. Inconclusive GC/CT NAAT was defined as presence of PCR amplification inhibitors using Roche Cobas v2.0 CT/NG assay. Clinical charts were abstracted for age, gender, HIV status, GC/CT (urogenital, rectal, pharyngeal) and syphilis screening results during the study period, clinic type (HIV clinic, university student health center, other), and whether repeat testing occurred within 6 months following an inconclusive result. Logistic regression analysis was used to calculate adjusted and unadjusted odds ratios of factors associated with receipt of repeat testing following an inconclusive rectal GC/CT NAAT result.

**Results:**

During the study period, 6.1% (852/14,015) of rectal GC/CT NAAT were inconclusive for one or both of GC and CT. Among the 413 patients whose inconclusive rectal GC/CT NAAT results that were included in our analysis, 66.6% (275/413) received repeat testing within 6 months, of which 8.7% (24/275) were positive (compared to 5.4% positivity rate of all rectal samples). In multivariable analysis, individuals living with HIV had lower odds of receiving repeat testing following inconclusive rectal GC/CT NAAT compared to HIV uninfected individuals (adj OR 0.25; p = 0.001).

**Conclusions:**

Despite being disproportionately affected by the STI epidemic, individuals living with HIV had 75% lower odds of receiving repeat testing following inconclusive rectal GC/CT NAAT compared to HIV-uninfected individuals, representing potentially missed opportunities for treatment and prevention of ongoing STI transmission.

## Introduction

Incidence of sexually transmitted infections (STIs) have been steadily increasing in recent years, with the two most prevalent bacterial STIs being *Neisseria gonorrhoeae* (GC) and *Chlamydia trachomatis* (CT) [1]. Appropriate screening and timely treatment are crucial to early interruption of the infection cycle, and prevent ongoing GC and/or CT (GC/CT) transmission, particularly among men who have sex with men (MSM) and HIV-infected individuals [1, 2]. Extragenital (rectal and pharyngeal) GC/CT infections are common among MSM and are frequently asymptomatic [3], with estimates that over 70% of extragenital GC infections and 85% of extragenital CT infections are missed with urethral screening alone [4]. As such, current CDC guidelines recommend screening for all sites of sexual contact for MSM at least annually and up to every 3–6 months based on risk behavior [2].

Nucleic acid amplification tests (NAAT) are commonly used for extragenital screening, as they are more sensitive than culture for detection of both GC and CT [5]. However, presence of nucleic acid amplification inhibitors (such as heme from blood, bile from feces, and urea in urine) can produce inconclusive results [6]. While inconclusive NAAT from inhibited nucleic acid amplification is well-described in the clinical microbiology literature [6–8], there is a paucity of data evaluating clinical practices surrounding inconclusive NAAT, specifically about retesting behaviors following an inconclusive result. Since inconclusive GC/CT screening can result in diagnosis and treatment delays, understanding factors associated with repeat testing is crucial to the development of effective screening programs and implementation of interventions to reduce incidence. This analysis aims to describe factors associated with repeat testing following inconclusive rectal GC/CT NAAT among individuals who underwent GC/CT screening at a large academic institution in Los Angeles.

## Materials and methods

### Study population and design

This is a retrospective chart review of individuals who received an inconclusive rectal GC/CT NAAT result at a single academic institution located in Los Angeles, California from whose samples were collected from 3/2016 to 6/2018. Clinical reports of laboratory record results were obtained for all individuals who received rectal GC/CT screening using NAAT during the study period at an institution that utilizes the Roche Cobas CT/NG v2.0 assay (Roche Diagnostics, Indianapolis, Indiana) for all GC/CT NAAT, which has an estimated sensitivity of 75.0–100% for rectal GC and 87.1–92.3% sensitivity for rectal CT [9, 10]. Inconclusive GC/CT was defined as the presence of PCR inhibitors resulting in an inability to amplify more than 20 copies of the positive PCR control for GC and/or CT during the sample run [11]. All specimens with an inconclusive result underwent repeat testing per institutional protocol. Following two inconclusive NAAT results on the same specimen, the test was resulted as inconclusive and released to the ordering provider who was responsible for contacting the patient regarding their results and coordinating necessary treatment/follow-up.

During the study period, a total of 258,968 GC and CT NAAT were performed on specimens obtained at all anatomic sites. Of these tests, 88.4% (n = 228,819/258,968) were GC and CT NAAT on urogenital samples, 6.1% (n = 15,791/258,968) were GC/CT NAAT on pharyngeal samples, and 5.4% (n = 14,015/258,968) were GC/CT NAAT on rectal samples. Among urogenital tests during the study period, 1.3% (n = 3,089/228,819) were positive, 98.4% (n = 225,051/228,819) were negative, and 0.2% (n = 468/228,819) were inconclusive for GC and/or CT. Among pharyngeal samples, 3.0% (n = 477/15,791) were positive and 97.0% (n = 15,312/15,791) were negative for GC and/or CT. No pharyngeal samples resulted in an inconclusive GC and/or CT NAAT result. Among rectal samples, 5.4% (n = 756/14,015) tests were positive, 88.4% (n = 12,393/14,015) tests were negative, and 6.1% (n = 852/14,015) tests were inconclusive for GC and/or CT. As our analysis was limited to inconclusive rectal specimens, GC/CT tests that were performed on the same specimen were combined to reflect the number of inconclusive rectal swabs. As such, the 852 inconclusive rectal GC/CT tests during the study period correlated to 436 rectal swabs.

Clinical charts were abstracted for age, gender, HIV status, STI screening (GC/CT and syphilis) and whether repeat testing occurred within 6 months following the inconclusive rectal GC/CT result. The type of clinic where the rectal GC/CT screening was ordered was stratified into three categories: clinics providing predominately HIV-related care, a university student health center, and all other outpatient clinics. At all clinic sites, rectal specimens were predominantly patient-collected. GC/CT screening for an individual was considered positive if there was one or more positive GC and/or CT result from any anatomical site (rectal, urogenital, or pharyngeal) prior to the inconclusive result during the study period. Syphilis testing was considered positive with the presence of a positive rapid plasma reagin (RPR) titer with positive *Treponema pallidum* particle agglutination assay (TPPA) confirmation (if previous titer negative or unavailable) and/or a titer that was 4-fold increased from historical titers, resulted during the study period.

### Statistical analysis

Bivariate analysis was conducted utilizing chi-squared analysis for categorical predictors and Kruskal-Wallis tests for non-parametric, continuous predictors. Unadjusted odds ratios of patient-level characteristics associated with having inconclusive rectal GC/CT NAAT repeated were calculated with logistic regression (reference group: not having rectal GC/CT NAAT repeated). Multivariable logistic regression was used to evaluate factors associated with whether the inconclusive rectal GC/CT NAAT was repeated, controlling for age, gender, clinic type, HIV status, and STI screening results (GC/CT, syphilis). As complete case analysis was utilized, 23 swabs were excluded due to missing data, resulting in an analytic sample of 413. All analyses were conducted using Stata 15.1 (StataCorp, College Town, Texas). The study protocol was reviewed and approved by the Office of the Human Research Protection Program (OHRPP) at the University of California, Los Angeles (IRB# 18–001161).

## Results

Among the 413 samples that underwent rectal GC/CT NAAT and had an inconclusive result, the median age of the individual was 32 years (range 18–71), 96.4% were male (n = 398), 34.6% were HIV-positive (n = 143), 7.3% had syphilis (n = 30), and 40.0% had positive GC/CT NAAT (n = 165) during the study period. At the time of the inconclusive result, 5 swabs were positive for rectal GC/CT. Among the 5 swabs that were both inconclusive and positive rectal GC/CT, 3 of these swabs were repeated within 6 months. Most inconclusive results occurred among individuals who received testing at either the HIV-specific clinic (39.5%; n = 163) or student health center (44.6%; n = 184). 66.6% (275/413) of inconclusive results were repeated within 6 months, of which 74.9% (206/275) were negative, 8.7% (24/275) were positive, and 16.4% (45/275) were again inconclusive.

Individuals who had GC/CT NAAT repeated tended to be younger (median age 31 years; IQR 25–40) than those not retested (36.5; 23–54; p = 0.054). 72.4% of individuals who obtained repeat testing were HIV-uninfected (199/275), compared to 51.5% who were not retested (71/138; p<0.001). Syphilis testing did not differ between the two groups, with syphilis infection occurring in 7.6% (21/275) of those who underwent repeat testing versus 6.5% (9/138) who did not (p = 0.681). Descriptive statistics stratified by whether inconclusive results were repeated are in Table 1.

**Table 1:** Characteristics of individuals with inconclusive rectal *Neisseria gonorrhoeae* and/or *Chlamydia trachomatis* (GC/CT) nucleic acid amplification testing, stratified based on presence of repeat testing within 6 months following the inconclusive result, Los Angeles, California, 3/2016-8/2018 (N = 413).

Variable	Repeated (n = 275)	Not Repeated (n = 138)	p-value
Age	31 (25–40)	36.5 (23–54)	0.054
Gender			
Female	5 (1.8)	10 (7.3)	**0.005**
Male	270 (98.2)	128 (92.8)	
Clinic			
Student	133 (48.4)	51 (37.0)	0.084
HIV	100 (36.4)	63 (45.7)	
Other	42 (15.3)	24 (17.4)	
HIV			
Negative	199 (72.4)	71 (51.5)	**<0.001**
Positive	76 (27.6)	67 (48.6)	
RPR			
Negative	254 (92.4)	129 (93.5)	0.681
Positive	21 (7.6)	9 (6.5)	
GC/CT			
Negative	141 (51.3)	107 (77.5)	**<0.001**
Positive	134 (48.7)	31 (22.5)	

Results are presented as either Median (IQR) or n (%). *Note*: Bold indicates p-value <0.05

In unadjusted analysis, men compared to women had more than four-fold increased odds of having an inconclusive rectal GC/CT result repeated (unadjusted OR 4.22; p = 0.010). In multivariable regression analysis, individuals living with HIV had significantly lower odds of repeat testing than their HIV-uninfected counterparts (adjusted OR 0.25, p = 0.001). History of positive GC/CT screening during the study period had almost three-fold increased odds of being retested compared to individuals with negative GC/CT (adjusted OR 2.95, p<0.001). We did not find evidence that age, clinic type, and syphilis screening results were associated with repeat testing in our adjusted analysis. Unadjusted and adjusted analysis of factors associated with repeat GC/CT NAAT are in Table 2.

**Table 2:** Unadjusted and adjusted odds ratios of selected variables associated with receipt of repeat testing following inconclusive rectal *Neisseria gonorrhoeae* and/or *Chlamydia trachomatis* (GC/CT) nucleic acid amplification testing, Los Angeles, California, 3/2016-8/2018.

Variable	Unadjusted OR	95% CI	p-value	Adjusted OR	95% CI	p-value
Age	0.98	0.96–0.99	**0.002**	0.99	0.97–1.02	0.613
Gender						
Female	Ref	Ref	Ref	Ref	Ref	Ref
Male	4.22	1.41–12.60	**0.010**	5.48	1.72–17.43	**0.004**
Clinic						
Student	Ref	Ref	Ref	Ref	Ref	Ref
HIV	0.61	0.39–0.96	**0.031**	1.96	0.82–4.69	0.129
Other	0.67	0.37–1.22	0.190	0.99	0.47–2.10	0.986
HIV						
Negative	Ref	Ref	Ref	Ref	Ref	Ref
Positive	0.40	0.26–0.62	**<0.001**	0.25	0.11–0.56	**0.001**
RPR						
Negative	Ref	Ref	Ref	Ref	Ref	Ref
Positive	1.19	0.53–2.66	0.681	1.62	0.65–4.02	0.299
GC/CT						
Negative	Ref	Ref	Ref	Ref	Ref	Ref
Positive	3.28	2.06–5.22	**<0.001**	2.95	1.81–4.80	**<0.001**

*Note*: Bold indicates p-value <0.05

## Discussion and conclusions

To the best of our knowledge, this is the first study evaluating factors associated with repeat rectal GC/CT testing following an inconclusive NAAT result. Among individuals with inconclusive rectal GC/CT NAAT, two-thirds underwent repeat testing within 6 months. Individuals who received repeat testing following inconclusive GC/CT NAAT had a higher positivity rate for GC and/or CT (8.7%) compared to all rectal samples screened during the study period (5.4%), demonstrating the importance of repeat testing following an inconclusive result. Individuals with a history of positive GC/CT screening during the study period had almost three-fold higher odds of having an inconclusive result repeated compared to those with negative GC/CT screening. These findings suggest that providers and/or patients are more active in pursuing repeat testing when the patient has a known history of GC/CT infection.

HIV-infected individuals had approximately 75% lower odds of receiving repeat testing following an inconclusive result compared to HIV-uninfected individuals. These results parallel previous literature describing suboptimal GC/CT screening among HIV-infected persons, with annual GC/CT screening rates for at least one anatomical site estimated at 20–39% [12–14] and even lower estimated rates for rectal GC/CT screening at 9% [15]. These findings are particularly concerning as HIV-infected individuals are disproportionately affected by the current bacterial STI epidemic compared to those not living with HIV [1], highlighting the continued need for improved screening efforts to facilitate timely treatment and prevention of STI transmission. Additionally, effective GC/CT screening is imperative to prevent HIV transmission, given known associations between GC/CT infection and HIV susceptibility, with a recent modelling study estimating that as much as 10% of incident HIV infections are attributable to GC/CT infection [16].

This study does have limitations. As this study took place at a single, large academic center in Los Angeles, our findings may not be generalizable to other settings. Additionally, this analysis was unable to evaluate reasons for why repeat GC/CT screening did not occur (e.g., testing was ordered but the patient did not return to get rescreened, retesting was pursued at an outside clinic, the patient had already received treatment for GC/CT at the time of inconclusive testing, etc.), as this information was not collected. Finally, while the FDA recently granted clearance of the Aptima Combo 2 (Hologic, Marlborough, Massachusetts) and Xpert CT/NG (Cepheid, Sunnyvale, California) NAAT assays for extragenital GC/CT screening, the Roche Cobas CT/NG assay (utilized in this study) remains uncleared by regulatory authorities [17]. However, as the Cobas CT/NG assay for GC/CT testing is widely used, and inconclusive test results can occur regardless of platform, our findings are relevant to any setting that utilizes NAAT for GC/CT screening, regardless of the assay.

While NAAT is widely used for GC/CT screening, a small but clinically significant proportion of samples tested may produce an inconclusive result. Despite being disproportionately affected by the STI epidemic, HIV-infected individuals were less likely to undergo repeat testing following inconclusive rectal GC/CT NAAT than HIV-uninfected individuals, representing potentially missed opportunities for treatment and prevention of ongoing STI transmission. Furthermore, samples from individuals who underwent repeat testing had a higher positivity rate for GC and/or CT compared to all rectal samples screened during the study period. Interventions to make providers aware of the recommendation for repeat testing when faced with an inconclusive result are needed.
